# Reduced Graphene Oxide/Au Nanocomposite for NO_2_ Sensing at Low Operating Temperature

**DOI:** 10.3390/s16071152

**Published:** 2016-07-22

**Authors:** Hao Zhang, Qun Li, Jinyu Huang, Yu Du, Shuang Chen Ruan

**Affiliations:** 1 Shenzhen Key Laboratory of Laser Engineering, College of Optoelectronic Engineering, Shenzhen University, Shenzhen 518060, China; haozhang13@sina.com; 2 Key Laboratory of Optoelectronic Devices and Systems of Ministry of Education and Guangdong Province, College of Optoelectronic Engineering, Shenzhen University, Shenzhen 518060, China; 3 Shenzhen Key Laboratory of Sensor Technology, College of Physics Science and Technology, Shenzhen University, Shenzhen 518060, China; onlyforbear@126.com (Q.L.); kingofdk@sina.com (J.H.)

**Keywords:** graphene, nanocomposite, NO_2_ sensing, low operating temperature

## Abstract

A reduced grapheme oxide (rGO)/Au hybrid nanocomposite has been synthesized by hydrothermal treatment using graphite and HAuCl_4_ as the precursors. Characterization, including X-ray diffraction (XRD), Raman spectra, X-ray photoelecton spectroscopy (XPS) and transmission electron microscopy (TEM), indicates the formation of rGO/Au. A gas sensor fabricated with rGO/Au nanocomposite was applied for NO_2_ detection at 50 °C. Compared with pure rGO, rGO/Au nanocomposite exhibits higher sensitivity, a more rapid response–recovery process and excellent reproducibility.

## 1. Introduction

Since air pollution has become an urgent global problem with the development of industry and technology, detecting gases, especially toxic gases, as the basis for controlling air pollution, has become increasingly significant. NO_2_ is a toxic compound produced by combustion in power plants and combustion engines. This gas is harmful to the environment and is a major cause of acid rain, photochemical smog and pollution haze. Up to now, metal oxides (MOS) semiconductor sensors have been widely used in NO_2_ sensing. In most cases, the operating temperature of MOS sensors is over 200 °C, which creates obstacles for fabricating integrated circuits and increases the energy consumption. Recently, a range of techniques such as surface functionalization with novel metals including Pd, Pt and Au or doping with novel metals [1,2,3,4,5,6,7], MEMS fabrication [8], nano-sensing materials [9], application of electrostatic fields [10], and ultraviolet (UV) irradiation have been developed to reduce the operating temperature and improve the sensitivity and stability of gas sensors [11,12,13,14,15,16,17,18].

Graphene, known as “the thinnest material in our universe” with only one-atom thickness, has attracted huge attention for its high electron mobility since its discovery. Because of its unique features of high surface area, light weight, high electron mobility and mechanical strength, graphene represents a very promising platform to load metal or semiconductor nanoparticles, organic and biological molecules for photocatalytic, optoelectronic, cellular imaging, and biosensor applications [19,20,21,22]. Due to its 2D structure, graphene can adsorb highly sensitive molecules which treat every carbon atom as a surface atom. Among the different methods to prepare graphene, chemical and thermally reduced graphene oxide (rGO) derived from the graphene oxide (GO) process based on Hummers’ method is mostly used [23]. Due to electrostatic repulsion of the versatile oxygen-containing groups (OCGs), an aqueous colloidal dispersion of GO can be used as the starting support material for fabricating advanced graphene-based nanomaterials [24]. Reduced graphene oxide is expected to be a promising sensing material due to its semiconductor properties. To date, although rGO has a relatively weak response compared to SnS_2_ with similar 2D structure [25], it could detect gases at low operating temperatures, even room temperature. 

In this work, a rGO/Au nanocomposite was synthesized through a facile one-step hydrothermal method. Sensors based on the rGO/Au and rGO were also fabricated, and their NO_2_ sensing performance was investigated. The rGO/Au exhibited p-type semiconductor behavior in the gas sensing process. Investigations on the gas sensors showed that sensors based on rGO/Au composites exhibited shorter response and recovery times compared with those of pure rGO at low operating temperature. Furthermore, a possible sensing mechanism for the detection of NO_2_ is also discussed.

## 2. Materials and Methods

### 2.1. Chemicals

All chemicals were of analytical grade and were used as received without further purification. Graphite and HAuCl_4_, were supplied by Beijing Chemical Corp, Ltd. (Beijing, China). The water used throughout all experiments was purified through a Millipore system (Millipore, Bedford, MA, USA).

### 2.2. Preparation of GO

GO was prepared from natural graphite powder through a modified Hummers’ method [26]. In a typical synthesis, 1 g of graphite was added into 23 mL of H_2_SO_4_, followed by stirring at room temperature for 24 h. After that, 100 mg of NaNO_3_ was introduced into the mixture and stirred for 30 min. Subsequently, the mixture was kept below 5 °C by ice bath, and 3 g of KMnO_4_ was slowly added into the mixture. After being heated to 35–40 °C, the mixture was stirred for another 30 min. 46 mL of water was then added into above mixture during a period of 25 min and the mixture was heated to 95 °C under stirring for 15 min. Finally, 140 mL of water and 10 mL of H_2_O_2_ were added into the mixture to stop the reaction. After the unexploited graphite in the resulting mixture was removed by centrifugation, as-synthesized GO was dispersed into individual sheets in distilled water at a concentration of 1 mg/mL with the aid of ultrasound for further use.

### 2.3. Preparation of rGO/Au Nanocomposite

rGO/Au composite was prepared by in situ production of Au nanoparticles on the surface of GO. In a typical synthesis, 0.5 mL of GO (1 mg/mL) were added into 20 mL of deionized water, followed by stirring for 10 min to get homogeneous yellow-brown colloidal. Then HAuCl_4_ (0.01 M) and sodium citrate was introduced into the GO solution, which was sonicated for 40 min. After that, further sonicating for 30 min, the aqueous dispersion was transferred into a 40 mL Teflon-lined, stainless-steel autoclave and heated at 180 °C for 12 h. The black product was harvested by centrifugation and washed with water and ethanol several times, and dried at 60°C for 12 h. For comparison, the rGO was prepared by the similar method without addition of HAuCl_4_.

### 2.4. Characterizations

Powder X-ray diffraction (XRD) data were recorded on a D/Max-2550 diffractometer (Rigaku, Tokyo, Japan) with Cu-Kα radiation (λ = 0.15418 nm). The transmission electron microscopic (TEM) images were performed on a JEM-3010 TEM microscope (JEOL, Tokyo, Japan) with an accelerating voltage of 200 kV. The sample for TEM characterization was prepared by placing a drop of colloidal solution on carbon-coated copper grid and dried at room temperature. X-ray photoelectron spectroscopy (XPS) analysis was measured on an ESCALABMK IIX-ray photoelectron spectrometer using Mg as the mexciting source. Raman spectra were obtained on a J-YT64000 Raman spectrometer with 514.5 nm wavelength incident laser light.

### 2.5. Fabrication and Gas Sensing Measurements

**Figure 1:**
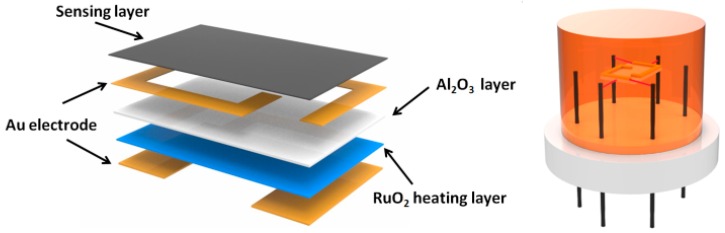
A schematic illustration of the sensor coated with the sensing material.

The prepared rGO/Au nanocomposite was mixed in a mortar with deionized water to obtain solution. Then the droplet has placed on a ceramic plate (1 mm × 1.5 mm), which was previously covered with gold electrodes and ruthenium oxides as heater on frontal and back sides by screen printing technique, followed by dryness at room temperature. Figure 1 shows a schematic illustration of the sensor coated with the sensing material. The response of the gas sensor is defined as the ratio of the resistance of the sensor in air (R_a_) to that in the tested gases (R_g_). For oxidizing tested gases, the expression is Response = R_a_/R_g_, while for the reducing tested gases, it is Response = R_g_/R_a_. The time taken by the sensor to achieve 90% of the total resistance change was defined as the response time in the case of adsorption or the recovery time in the case of desorption. The gas-sensing properties of sensors were measured using a CGS-8 gas-sensing characterization system.

## 3. Results

### 3.1. Structural and Morphological Characteristics

**Figure 2:**
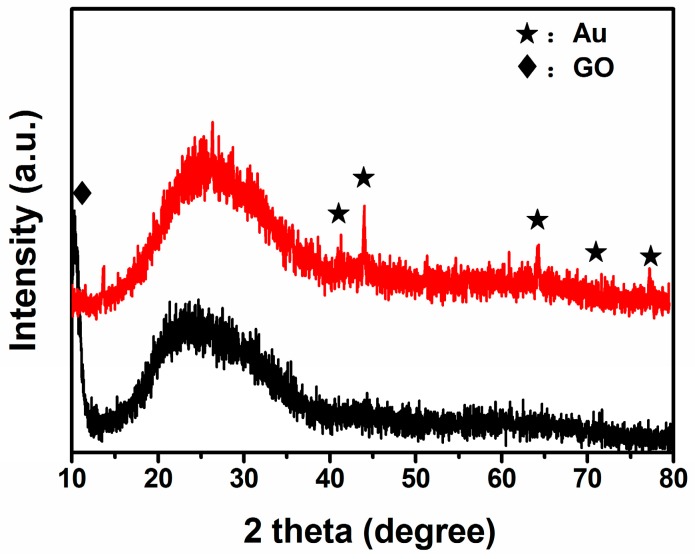
The XRD patterns of GO (black line) and rGO/Au (red line).

The phase composition of the hybrid structures is analyzed by XRD. A strong peak at 2θ of 11.26° corresponding to the (002) interlayer d spacing of 7.85 Å is observed in Figure 2, indicating the successful preparation of GO by oxidation of graphite [27]. However, no diffraction peaks can be ascribed to graphene, probably due to self-reassembling of exfoliated graphite oxide or the reduction of GO by hydrothermal treatment [28,29]. Furthermore, several peaks are observed at 43.1°, 44°, 64.2°, 70.1°, 77.2° which are attributed to Au nanoparticles (JCPDS card No. 01-1172) indicating the formation of Au crystals in the composite material.

**Figure 3:**
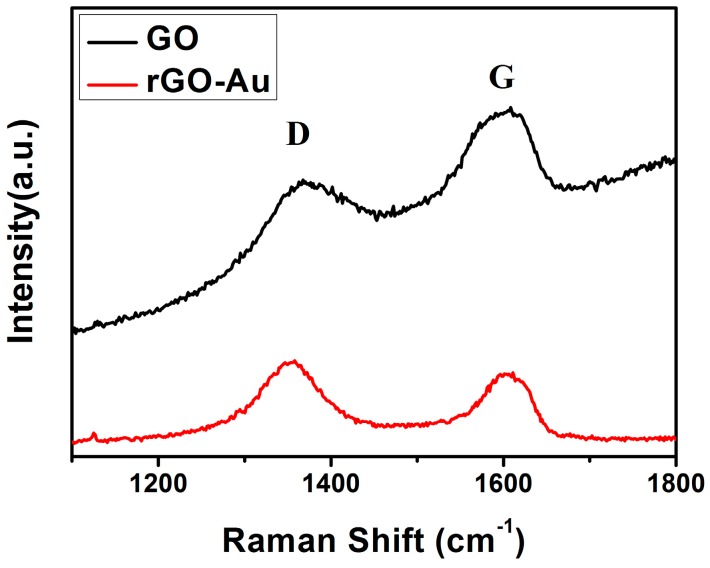
Raman spectroscopy of the GO (black line) and rGO/Au (red line) samples.

Figure 3 shows the Raman spectra confirming the reduction of GO. Two strong peaks of the D band (~1351 cm^−1^) and G band (~1595 cm^−1^) were observed, which correspond to the diamondoid and graphitic graphene structures, respectively. It is well known that the intensity ratio of the D and G band (ID/IG) is strongly related to the quantity of functional groups of rGO, and compared with ID/IG value of GO (0.796), the increased value of rGO (1.131) suggests restoration of C=C bonds after hydrothermal reduction [30].

**Figure 4:**
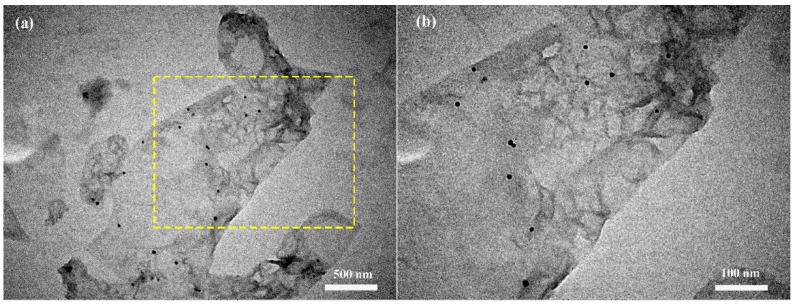
(**a**) A TEM images of rGO/Au; (**b**) An enlarged image of selected area.

The typical TEM image of rGO/Au is shown in Figure 4. It is clearly seen that the rGO has been decorated with a few amount of Au nanoparticles in Figure 4a. Almost all the Au nanoparticles are distributed uniformly on the rGO surface and have a size of about 10 nm (Figure 4b). The presence of isolated Au nanoparticles reveals that hydrothermal treatment of GO and HAuCl_4_ solution is an effective method for the preparation of rGO/Au nanocomposite.

**Figure 5:**
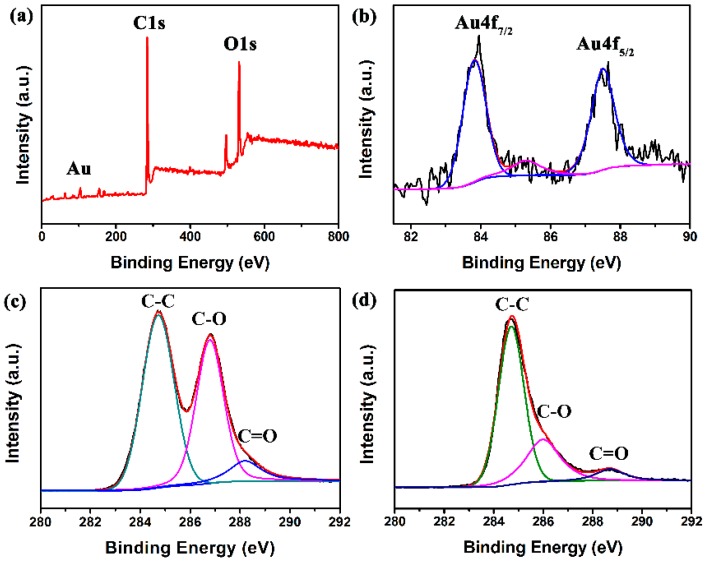
(**a**) XPS spectras of rGO/Au; (**b**) Au4f spectrum of rGO/Au; (**c**) C1s spectrum of GO; (**d**) C1s spectrum of rGO/Au.

To further investigate the characteristics of the products, XPS technique was used to analyze the chemical state of GO and rGO/Au samples. Figure 5a shows the XPS spectrum of rGO-Au composite. Two peaks at about 284.6 eV and 532.0 eV are observed, which are attributed to C1s and O1s bands, respectively. Note that two significant signals at 83.3 and 86.9 eV, and a miner peak at 85.3 eV are exhibited corresponding to metallic Au and Au(I), respectively (Figure 5b), which further confirms the presence of Au element in the final samples. The intensity of the Au4f signal is higher than that of Au(I), indicating that most of Au is of zero valence and exits in the metallic form. Figure 5c,d reveal the C1s spectra of GO and rGO/Au samples, exhibiting three peaks at 284.6, 286.6 and 288.4 eV, attributed to the C–C, C–O and C=O bands in graphene-based materials [31]. It is worth noting that compared to the peak intensity of C–O and C=O in GO, those of rGO–Au decrease tremendously, which suggests that most oxygen-containing functional groups were successfully removed after hydrothermal treatment. All these observations indicate the successful formation of Au and reduction of GO by hydrothermal reaction of GO and HAuCl_4_.

### 3.2. NO_2_ Sensing Properties

**Figure 6:**
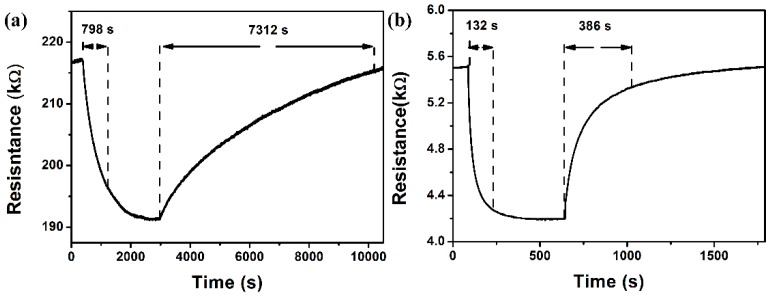
The response curve to 5 ppm NO_2_ of the sensors based on (**a**) rGO; (**b**) rGO/Au at 50 °C.

Figure 6 shows the response and recovery times of sensors based on the rGO and rGO/Au to 5 ppm NO_2_, respectively. The response time, T_r1_ value (recovery time, T_r2_ value) of rGO/Au sensors upon exposure to 5 ppm NO_2_ gas is 132 s (386 s) at a low operating temperature of 50 °C (Figure 6b). Compared with that, the pristine rGO sensor exhibits a longer T_r1_ (T_r2_) of 798 s (7312 s) in Figure 6a. It is concluded that loading Au nanoparticles is an effective method for shortening response/recovery time (T_r1_/T_r2_) for graphene-based gas sensors at a low operating temperature. Additionally, It is found that rGO/Au sensor exhibits remarkably enhanced response of 1.33 to 10 ppm NO_2_, which has been improved up from 1.13 from pure graphene sensor.

**Figure 7:**
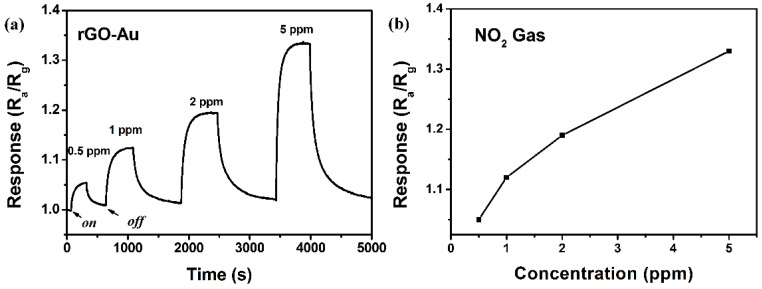
(**a**) Dynamic NO_2_ sensing transients curve of the rGO/Au-based sensor to 0.5–5 ppm NO_2_ at 50 °C; (**b**) The responses of the rGO/Au based sensor to 0.5–5 ppm NO_2_ at 50 °C.

Figure 7a shows the response of the rGO/Au based sensor being orderly exposed to 0.5–5 ppm NO_2_ gases at a low operating temperature of 50 °C. It is shown that the response and recovery characteristics are in agreement with the above analysis of Figure 6b. Moreover, it is also found that the response gradually rises as the concentration of NO_2_ increases, showing a range of linear response (Figure 7b). The response values of the sensor to 0.5, 1, 2 and 5 ppm NO_2_ gas are about 1.05, 1.12, 1.19 and 1.33, respectively.

**Figure 8:**
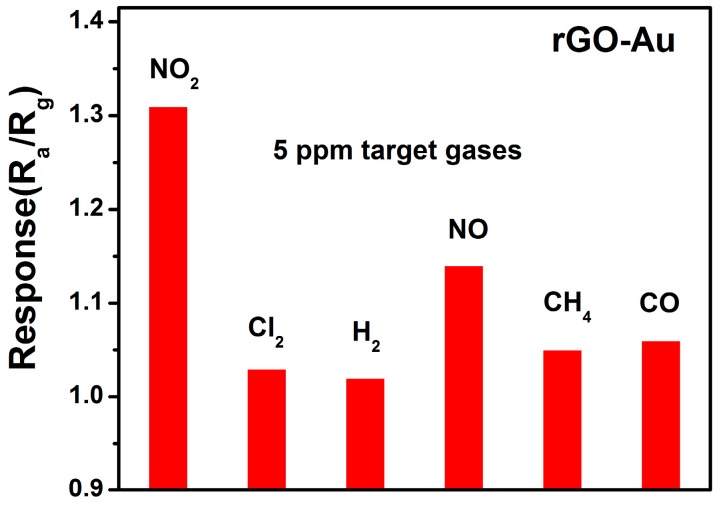
The responses of rGO/Au based sensor to 5 ppm of different gases at 50 °C.

The selectivity of the gas sensor is also important for practical application. Figure 8 shows the responses of rGO/Au based sensor to potential interference gases (Cl_2_, H_2_, NO, CH_4_, CO). Take 5 ppm target gases for example, the sensor only gives a relatively lower response to interference gases, indicating the sensor has a good selectivity.

**Figure 9:**
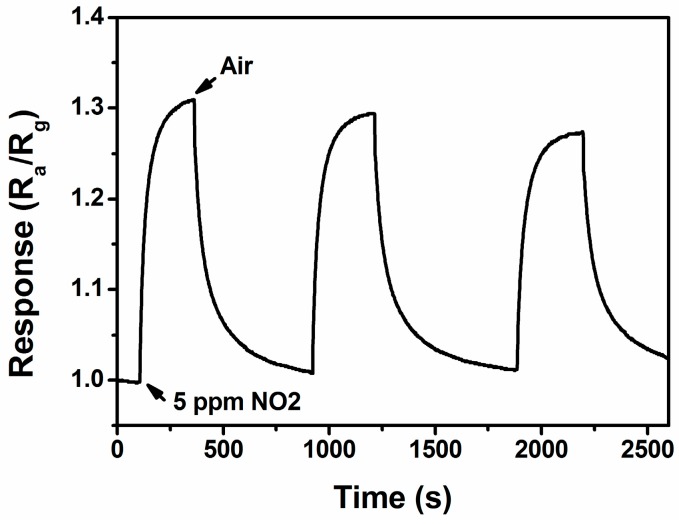
The reproducibility of the rGO/Au sensor on successive exposure (3 cycles) to 5 ppm NO_2_ at 50 °C.

The further test for repeatability of the sensor based on rGO/Au is illustrated in Figure 9. It is revealed that the sensor maintains its initial response amplitude without a clear decrease upon three successive sensing tests to 5 ppm of NO_2_, albeit the swift response and recovery process, indicating that the sensor has an outstanding repeatability throughout the cycle test.

## 4. Gas Sensing Mechanism of rGO/Au

It is well shown that the pure rGO exhibits a relatively weak response and long response and recovery times for detection of NO_2_ at 50 °C. The unsatisfactory sensitivity of the rGO sensor results from its constituent carbon atoms. There are two types of carbon atoms in graphene, sp^2^ hybridized carbon atoms constituting the graphite structure, and sp^3^ hybridized carbon atoms constituting structural defects and forming a chemical bond with oxygen-containing groups. The adsorption energy of the latter is larger (5.7 kcal/mol), resulting in slower adsorption and desorption [32,33].

**Figure 10:**
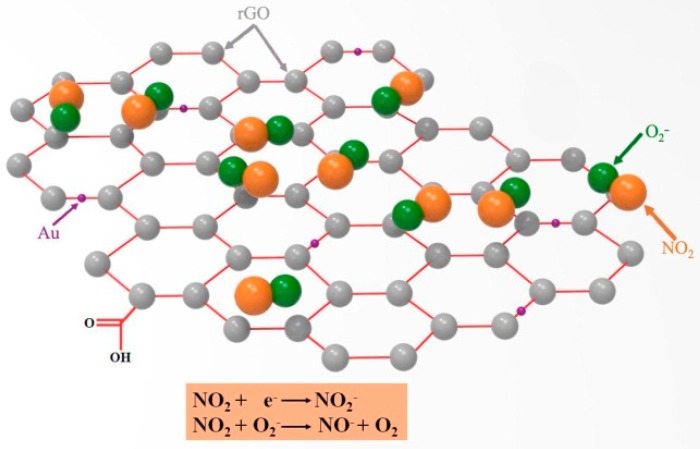
The scheme of the proposed gas sensing mechanism: the adsorption behavior of NO_2_ molecules on the rGO/Au nanocomposite.

The improvement of NO_2_ gas sensing properties of rGO nanosheets by Au-functionalization can be explained as follows: firstly, the principle of gas sensing for the resistance-type sensors is based on the conductance variations of the sensing element, thus the introduction of Au to rGO contributes significantly to improving the conductivity, leading to a better sensing behavior. Secondly, based on the model proposed for the metal catalyst-enhanced gas sensing of nanomaterials [34], the NO_2_ gas was spilt over the rGO nanosheet surface by Au nanoparticles, and the chemisorption and dissociation of NO_2_ gas was enhanced on the Au nanoparticle surface due to the high catalytic or conductive nature of Au. Consequently, the number of electrons attracted to the gas increases. Thirdly, the electron transfer from the defect states to the Au nanoparticles not only results in an increase in resonant electron density, but also creates energetic electrons in high energy state [35,36]. These resonant electrons are so active that they can escape from the surface of Au nanoparticles to the NO_2_. As shown in Figure 10, the role of Au as an electron mediator further facilitates the electron transfer from rGO to NO_2_ molecules. Therefore, the electron density rGO decreased significantly by Au-functionalization.

## 5. Conclusions

The synthesis of well controlled rGO/Au composites by hydrothermal treatment is demonstrated. The gas sensing results showed that the rGO/Au sensors could detect NO_2_ gas at levels as low as 0.5 ppm. The sensitivity of rGO/Au to 5 ppm NO_2_ (1.33) is higher than that of rGO (1.13). Moreover, the response (recovery) time towards 5 ppm NO_2_ improved from 798 s (7312 s) to 132 s (386 s) via the introduction of the Au nanoparticles. All results indicate that Au nanoparticle loading can significantly enhance the NO_2_ sensing properties of graphene-based sensing materials at a low operating temperature, which indicates excellent potential applications as gas sensors.
